# Development of an Anti-Idiotype Aptamer-Based Electrochemical Sensor for a Humanized Therapeutic Antibody Monitoring

**DOI:** 10.3390/ijms24065277

**Published:** 2023-03-09

**Authors:** Madoka Nagata, Jinhee Lee, Taro Saito, Kazunori Ikebukuro, Koji Sode

**Affiliations:** 1 Joint Department of Biomedical Engineering, The University of North Carolina at Chapel Hill and North Carolina State University, Chapel Hill, NC 27599, USA; mnagata@email.unc.edu (M.N.); jinc0324@gmail.com (J.L.); 2 Department of Biotechnology and Life Science, Graduate School of Engineering, Tokyo University of Agriculture and Technology, Koganei 184-8588, Japan; hanataro191110@gmail.com (T.S.); ikebu@cc.tuat.ac.jp (K.I.)

**Keywords:** aptamers, bevacizumab, biosensors, therapeutic monoclonal antibody, point-of-care testing

## Abstract

Therapeutic monoclonal antibodies (mAbs) are currently the most effective medicines for a wide range of diseases. Therefore, it is expected that easy and rapid measurement of mAbs will be required to improve their efficacy. Here, we report an anti-idiotype aptamer-based electrochemical sensor for a humanized therapeutic antibody, bevacizumab, based on square wave voltammetry (SWV). With this measurement procedure, we were able to monitor the target mAb within 30 min by employing the anti-idiotype bivalent aptamer modified with a redox probe. A fabricated bevacizumab sensor achieved detection of bevacizumab from 1–100 nM while eliminating the need for free redox probes in the solution. The feasibility of monitoring biological samples was also demonstrated by detecting bevacizumab in the diluted artificial serum, and the fabricated sensor succeeded in detecting the target covering the physiologically relevant concentration range of bevacizumab. Our sensor contributes to ongoing efforts towards therapeutic mAbs monitoring by investigating their pharmacokinetics and improving their treatment efficacy.

## 1. Introduction

Therapeutic monoclonal antibodies (mAbs) are currently the most effective medicines for a wide range of diseases because of their specificity and efficiency [1]. The first therapeutic mAb, muromonab-CD3, was approved by the U.S. Food and Drug Administration (FDA) in 1986. One of the problems of this therapeutic mAb was that it was derived from mice; proteins derived from mice lead to the production of antibodies against the administrated mAbs. This problem was solved by genetically engineering chimeric mAbs and humanized mAbs, which consist of a human-derived Fc region with the Fab region derived from mice. This engineering succeeded in reducing the unexpected immune response. Furthermore, the problem of immunogenicity can be said to be largely overcome with the development of fully humanized mAbs. The development of novel therapeutic mAbs is actively progressing, and the market for therapeutic mAbs is expected to continue its expansion, as in 2021 the 100th therapeutic mAb was approved by the FDA.

As in all pharmaceuticals, the therapeutic activity of mAbs is closely related to its concentration in serum. It is known that an insufficient concentration of therapeutic mAbs leads to the acquisition of drug resistance in cancer cells [2]. On the other hand, the administration of therapeutic mAbs at a concentration greater than necessary leads to unexpected side effects [2]. Therefore, appropriate administration is strongly needed to maintain the blood therapeutic mAbs concentrations within a stringent range. However, determining the appropriate administration timing for therapeutic mAbs is difficult because the individual pharmacokinetics of therapeutic mAbs are highly variable. The precise monitoring of therapeutic mAb levels by means of a biosensor could be an attractive method to circumvent this challenge.

To effectively analyze the concentration of therapeutic mAbs, it is necessary to specifically recognize the complementarity determining region (CDR) of their structures to distinguish them from immunoglobulins (IgGs), which are natively abundant in blood/serum. Quantitative MS analysis [3] and liquid chromatography-tandem mass spectrometry with immune affinity purification [4,5] were employed previously to measure the concentration of therapeutic mAbs from body fluid after administration. Other conventional methods that do not require large equipment are immunoassays, such as the enzyme-linked immunosorbent assay (ELISA) [6] and the electrochemiluminescence immunoassay (ECLIA) [7]. However, these immunoassays can only be performed in a central laboratory and may suffer from lower precision [8]. To realize the point-of-care (POC) testing of therapeutic mAbs, the development of therapeutic mAbs detection with handheld devices has been recently reported by employing partial sequences corresponding to epitopes or anti-idiotype antibodies. van Rosmalen et al. reported a bioluminescent platform (LUMABS) that allows for the detection of target mAbs from blood plasma directly using the camera of a smartphone [9], and Zeni et al. reported a surface plasmon resonance (SPR) based infliximab sensor using portable SPR devices [10].

Aptamers are single-stranded oligonucleotides with high target specificity which have been applied extensively as molecular recognition elements in biosensors [11,12]. DNA aptamers have numerous benefits over protein ligands in terms of thermal stability, ease of modification, cost-effectiveness, regenerability, and particularly the ease of development and synthesis [13,14]. One of the key benefits of using an aptamer as a recognition element is the flexibility of its structure. Electrochemical aptamer-based (E-AB) sensors are constructed to leverage an aptamer’s conformational flexibility to create signal change following target binding [15]. Since E-AB sensors do not require a chemical reaction nor free redox probe in the solution, they are very suitable for *in vivo* continuous monitoring. Another key benefit of aptamers is that they can be easily targeted to bind specific regions on the biomarker of interest [16,17]. A critical example of this is in the development of the anti-idiotype aptamer, which binds to the CDR of IgG and can thus distinguish the target IgG from other natural IgGs. The first anti-idiotype aptamer was reported as an RNA molecule binding to the antibody against the Rev-nuclear export signal (NES) [18]. We recently reported the development of an anti-idiotype aptamer that recognizes the CDR of bevacizumab [19]. Since anti-idiotype aptamers are able to distinguish target therapeutic mAbs from other natural IgGs, they are attractive molecular recognition candidates for use in biosensors fabricated for the monitoring of therapeutic mAb levels.

Bevacizumab is a humanized mAb which binds to vascular endothelial growth factor-A (VEGF-A). The FDA approved it as a therapy for the treatment of several types of cancers [20,21]. Although the mean half-life of bevacizumab has been reported as 20 days [22], several reports demonstrated that there is a large individual difference with half-lives ranging between 11 and 50 days [2]. Since the therapeutic activity of bevacizumab is closely related to its concentration in serum [2], the precise monitoring of bevacizumab is expected to improve its anti-tumor efficacy on an individual basis, while reducing the threat of any potential side effects. Our previously reported anti-idiotype aptamer which recognizes the CDR of bevacizumab was developed by the competitive systematic evolution of ligands by exponential enrichment, or SELEX. It was demonstrated that the developed aptamer, A14#1, could distinguish the target bevacizumab from human IgG [19]. We obtained a crystal structure of A14#1 in complex with bevacizumab, which revealed that this aptamer formed hydrogen bonds with several amino acids in the CDR. Using this aptamer, we previously reported bevacizumab detection based on an enzyme-linked aptamer assay and liquid chromatography [22,23]. An electrochemical detection system was also achieved using magnetic beads which were immobilized with anti-idiotype aptamer and alkaline phosphatase labeled IgG on carbon screen-printed electrodes [19]. However, this detection scheme required bound/free separation (B/F separation), and thus a sensing system that is further simplified will be required for the realization of a device suitable for the POC testing of bevacizumab. 

**Scheme 1:**
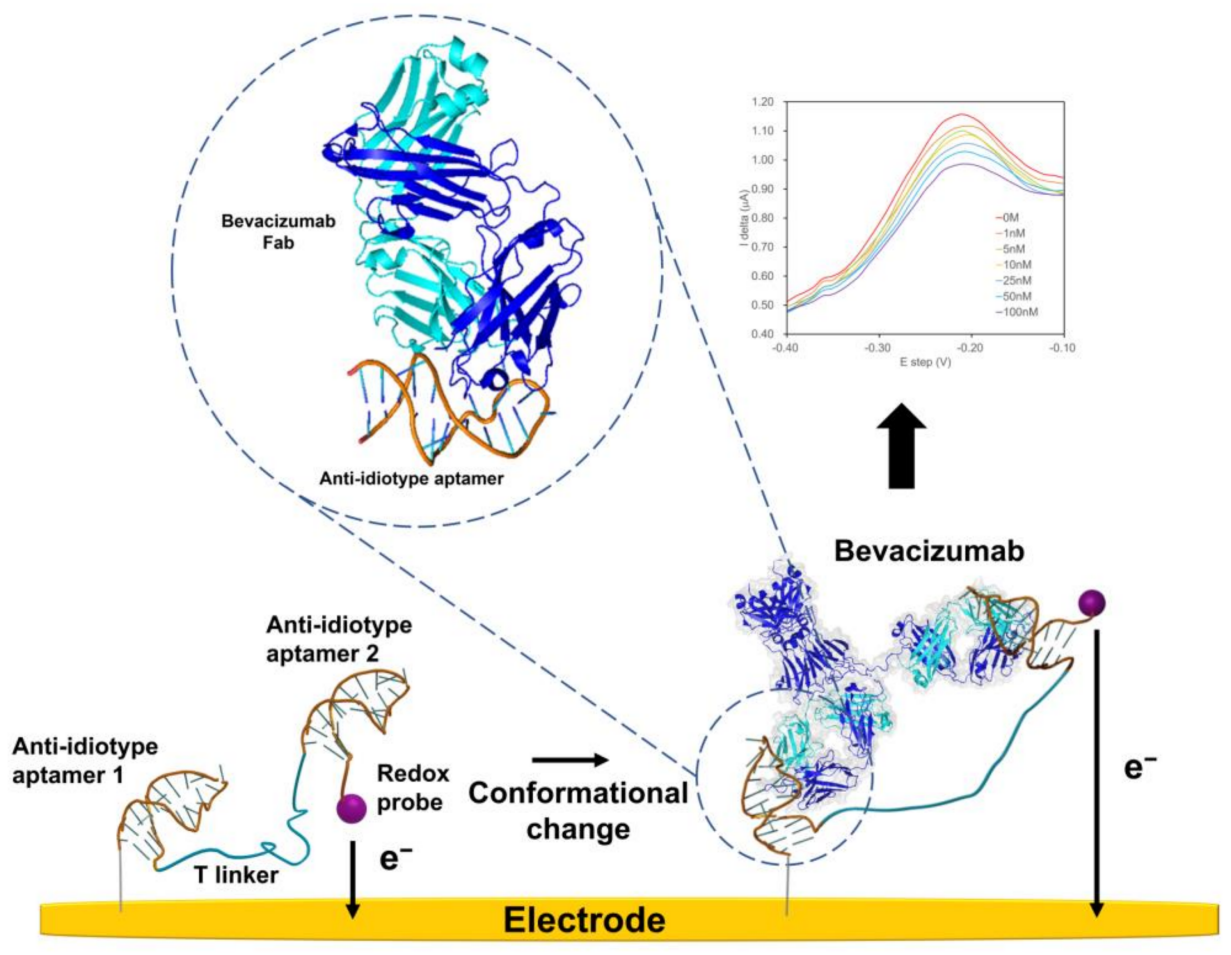
Schematic detailing the conformational change of a redox probe-modified bivalent anti-idiotype aptamer upon binding to bevacizumab. Two anti-idiotype bevacizumab aptamers (orange) connected via 44mer flexible thymine linker (T linker; cyan) bind to two CDRs of bevacizumab (PDB ID: 7V5N), consequently causing a large conformation change. This conformational change alters the electron transfer kinetics between the redox probe and the electrode surface depending on the bevacizumab concentration, which can be monitored by SWV.

This paper reports an electrochemical, anti-idiotype aptamer-based bevacizumab sensor, which will enable the development of a hand-held, rapid, sensitive POCT-type device which does not require B/F separation. We demonstrate the square wave voltammetry (SWV)-based bevacizumab sensing with redox probe-modified bivalent anti-idiotype aptamer as a biosensing molecule, consisting of two anti-idiotype aptamers binding to bevacizumab CDRs, which are linked with 44 mer flexible thymine linkers corresponding to the distance between two CDRs. The binding of two anti-idiotype aptamers to bevacizumab causes the drastic conformation change of the redox-probe modified bivalent anti-idiotype aptamer, resulting in the change of dynamics of accessing the redox probe on the electrode, consequently resulting in the change of peak current in the SWV measurement in the presence of bevacizumab in the sample solution. (Scheme 1). The sensor does not require a washing step and does not contain a free redox probe in the reaction solution. Further, considering the superb characteristic of the SWV-based aptamer sensor, we demonstrated the feasibility of monitoring bevacizumab in a biologically relevant sample matrix using an artificial serum solution. 

## 2. Results

### 2.1. Electrochemical Characterization of Anti-Bevacizumab Aptamer Immobilized Electrode

First, the anti-bevacizumab aptamer A14#1 immobilized electrode surface was characterized by electrochemical impedance spectroscopy (EIS). Bare electrodes showed a small semicircular Nyquist plot (Figure S1). After the modification of the anti-bevacizumab aptamer, both real and imaginary impedances were significantly increased. We attribute this observation to a reduction in the access of the redox probe to the electrode surface after self-assembled monolayer (SAM) formation. These results preliminarily suggest that the modification of the aptamer on the electrode surface was successful.

**Figure 1:**
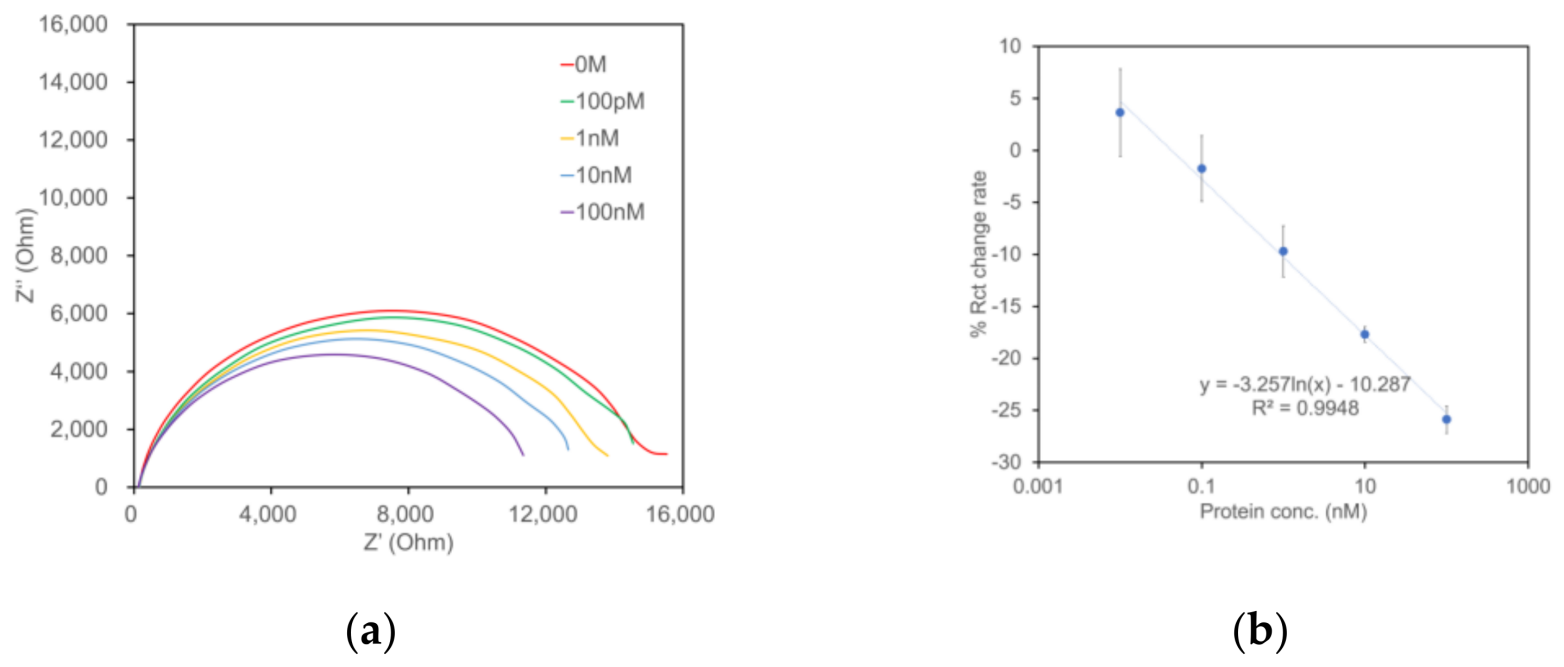
EIS monitoring of bevacizumab using the anti-bevacizumab aptamer A14#1 immobilized electrode. (**a**) is with the addition of bevacizumab. (**b**) is relative Rct change plotted against the bevacizumab concentration gradient.

To investigate the binding of bevacizumab to the anti-bevacizumab aptamer A14#1, which is immobilized on the electrode, the A14#1 immobilized electrode was incubated with bevacizumab or human IgG kappa chain (100 pM–100 nM) in 10 mM phosphate buffered saline (PBS) solution. With the addition of bevacizumab, the diameter of the Nyquist plot decreased according to the bevacizumab concentration (Figure 1a). On the contrary, the Nyquist plot with the addition of the human IgG kappa chain slightly increased (Figure S2). The charge transfer resistance (Rct) values, which are represented by the second resistor in parallel with the double-layer capacitance, were obtained by fitting to a Randles circuit. The normalized Rct values of the final concentration (Rct_f_) were calculated by the following equation:

$$\frac{{\mathrm{Rct}}_{f}-{\mathrm{Rct}}_{0M}}{{\mathrm{Rct}}_{0M}}$$

Rct change showed a high logarithmic correlation (R^2^ = 0.995) with the bevacizumab concentration from 100 pM to 100 nM (Figure 1b). These results indicate that the anti-bevacizumab aptamer A14#1 binds to bevacizumab on the electrode surface and can distinguish the target from other proteins. These results support the assumption that the electrode was successfully modified with anti-bevacizumab aptamer A14#1 while retaining its selectivity and sensitivity towards bevacizumab. 

### 2.2. Characterization of Bevacizumab Sensor Employing Redox Probe Modified Anti-Bevacizumab Aptamers 

We prepared electrodes immobilized with redox-probe modified anti-bevacizumab aptamer and subjected them for electrochemical investigation as a bevacizumab sensor based on SWV in PBS solution.

First, anti-bevacizumab aptamer A14#1 was applied for SWV-based sensing using electrodes immobilized with phenazine ethosulfate (PES)-modified A14#1. However, no peak current change was observed with the addition of bevacizumab (Figure S3). Considering that A14#1 kept its binding ability after the immobilization on the gold electrode, as was demonstrated above, we assumed that the binding of the A14#1 aptamer to bevacizumab did not provide enough conformation change to be detected by SWV. Therefore, we employed the bivalent A14#1 as an alternative anti-bevacizumab aptamer for the SWV based bevacizumab sensor after the modification by PES (PES-bivalent A14#1). Bivalent A14#1 consists of two A14#1 and 44 mer flexible thymine linkers, which corresponds to the distance of 2 CDRs [19]. Two proximate A14#1 can bind to two CDRs, therefore we considered that conformation change depending on the target binding would had occurred.

**Figure 2:**
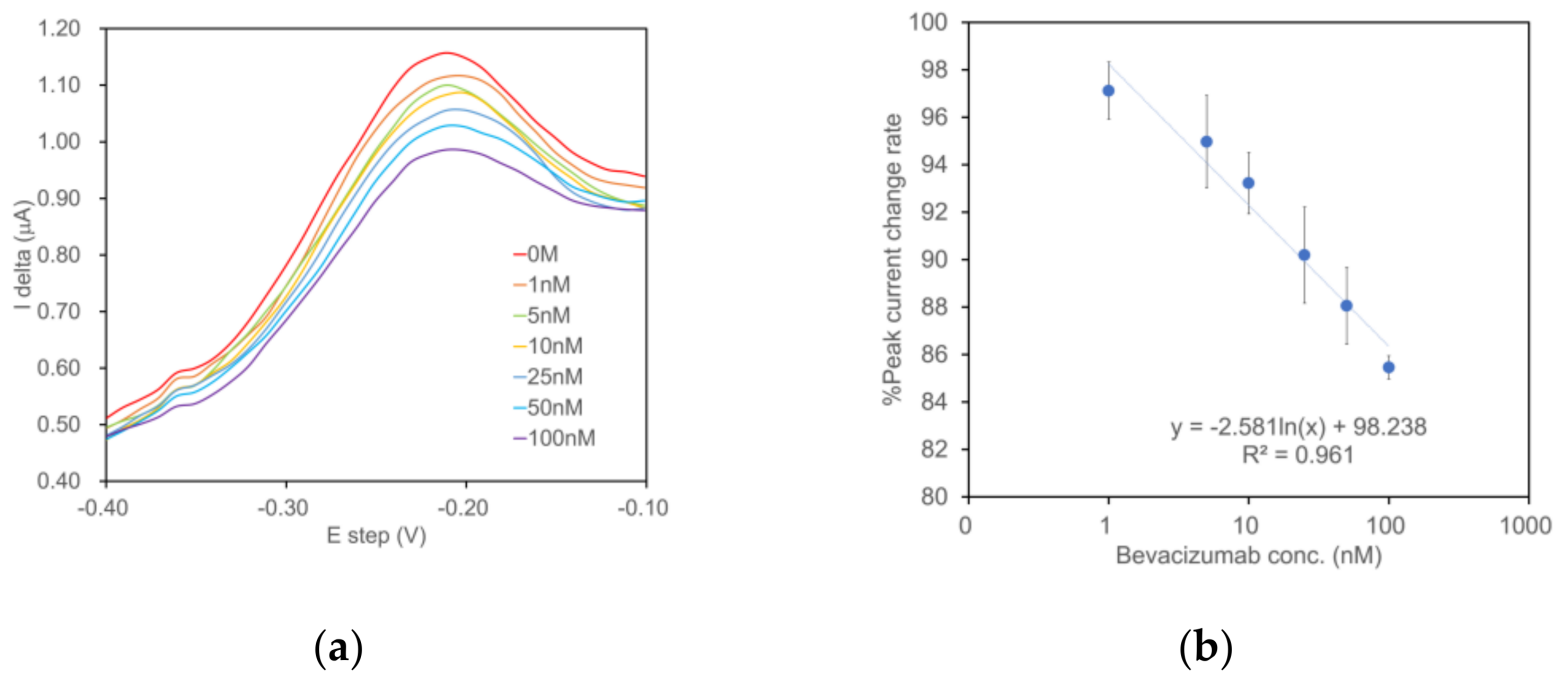
(**a**) Square-wave voltammogram of PES-bivalent A14#1 aptamer immobilized sensor at 100 Hz in PBS solution. SWV scans were conducted as the following conditions: −0.4 to −0.1 V, 25 mV amplitude. Seven concentrations of bevacizumab were used: 0 nM (the base line in the absence of bevacizumab),1, 5, 10, 25, 50, and 100 nM. (**b**) Concentration-response curve; the % of signal change calculated as 0M = 0%. The error bars indicate the standard deviations (*N* = 3).

The results of sensor responses toward an increasing concentration of bevacizumab are shown in Figure 2a. Electrodes immobilized with the PES-bivalent A14#1 aptamer showed a signal-off response in which the peak current decreased depending on the bevacizumab concentration. Since the highest signal change was observed at 100 Hz (Figure S4), we decided to use this frequency as the optimal frequency of bevacizumab detection. Figure 2a shows the square wave voltammogram of electrodes immobilized with the PES-bivalent A14#1 aptamer at 100 Hz in the presence of several concentrations of bevacizumab (1–100 nM). The peak current decreased depending on the concentration of bevacizumab. Figure 2b shows the correlation of peak current change rate and bevacizumab concentration with a dynamic range of 1–100 nM. A good linear correlation (R^2^ = 0.961) between the sensor signal change rate and logarithmic bevacizumab concentration was observed. The limit of detection (LOD) was calculated as 3SD/slope, where SD is the standard deviation of the peak current of the blank and the slope is from the calibration curve. The obtained LOD was 9.1 nM. The fabricated sensor detected bevacizumab in a concentration range between 1–100 nM without a washing step or free redox probes in the measurement solution.

**Figure 3:**
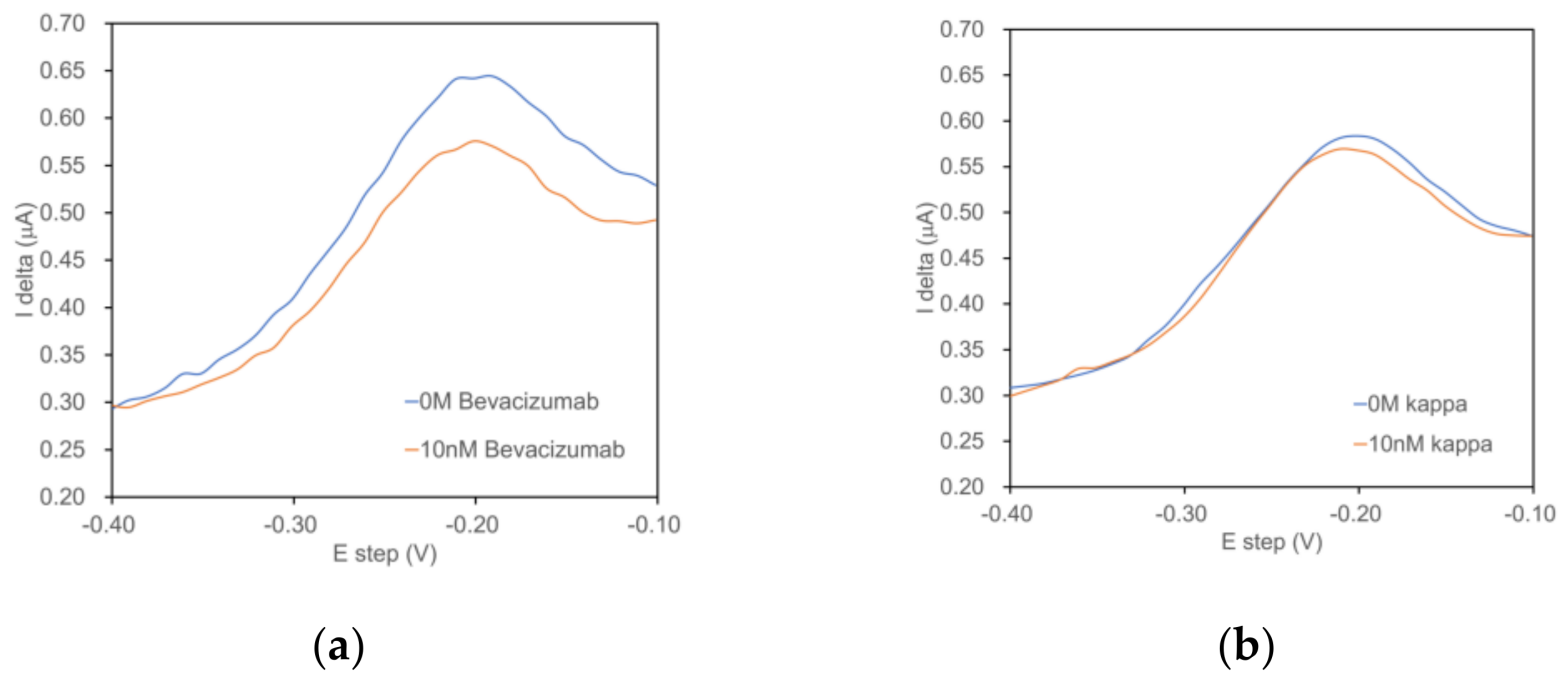
Square−wave voltammograms recorded with the addition of (**a**) 10 nM bevacizumab and (**b**) 10 nM human IgG kappa chain.

To test the selectivity of the SWV bevacizumab sensor constructed using the bivalent aptamer, a sample was spiked with either 10 nM bevacizumab or IgG kappa chain. With the addition of 10 nM of bevacizumab, the peak current of SWV decreased by 10% (Figure 3a), while, on the other hand, less than a 3% decrease was observed with the spiking of the same concentration of human IgG kappa chain (Figure 3b). Since the configuration of the aptamer-immobilized electrode is identical as the one investigated with the EIS method, the signal derived from IgG kappa should be due to the non-specific adsorption on the electrode. These results demonstrate that the SWV aptamer-based bevacizumab sensor can distinguish bevacizumab from other natural IgGs. Since the SWV-based aptasensor does not need a free redox probe in solution, it could be suitable in the future for in vivo monitoring.

### 2.3. Bevacizumab Detection from Artificial Serum Solution

**Figure 4:**
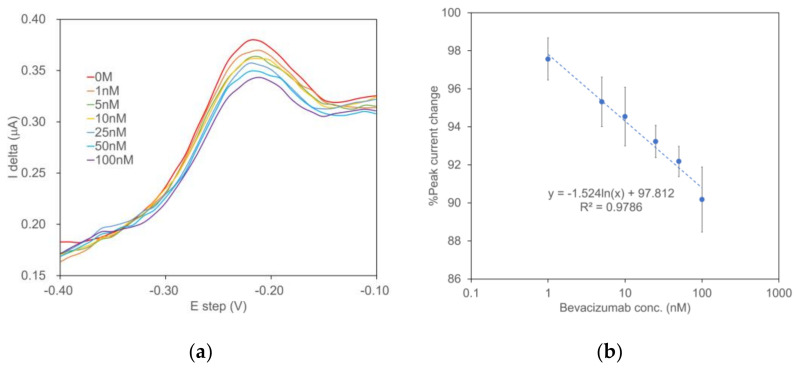
(**a**) Square−wave voltammograms of fabricated PES-bivalent A14#1 aptamer immobilized sensor at 100Hz in 10-fold diluted artificial serum solution. SWV scans were conducted according to the following conditions: −0.4 to −0.1 V, 25 mV amplitude. Seven concentrations of bevacizumab were used: 0 nM (the base line in the absence of bevacizumab), 1, 5, 10, 25, 50, and 100 nM. (**b**) Concentration-response curve; the % of signal change calculated as 0M = 0%. The error bars indicate the standard deviations (*n* = 3).

We decided to then evaluate the feasibility of the SWV-based bevacizumab sensor with the PES-bivalent A14#1 aptamer in a biologically relevant sample matrix. We employed a 10-fold diluted artificial serum that contains 53 μM BSA and 8 μM human IgG kappa chain as interferences. A concentration-dependent peak current decrease was observed at 100Hz in bevacizumab spiked artificial serum solution (Figure 4a). The fabricated sensor retained its ability to specifically bind bevacizumab and a good logarithmic correlation (R^2^ = 0.979) between the sensor signal change rate and bevacizumab concentration was observed (Figure 4b). The LOD was calculated as 3SD/slope, where SD is the standard deviation of the peak current of blank and the slope is from the calibration curve. The obtained LOD in artificial serum solution was 10.1 nM.

## 3. Discussion

In this paper, we reported the development of an electrochemical sensor for monitoring therapeutic mAb, bevacizumab, by employing a redox probe-modified anti-idiotype bivalent aptamer based on SWV, which can enable the development of a hand-held, rapid, sensitive POCT-type device which does not require B/F separation.

Anti-idiotype or anti-Fab aptamers have been reported as inhibitors against antibodies derived from their idiotypic properties [18,24,25], although the biosensor applications of the anti-idiotype aptamer were limited. In this study, we focused on the anti-idiotype aptamer as a highly selective biosensing molecule. In a previous report, the anti-idiotype bevacizumab aptamer A14#1 was developed and applied for electrochemical sensing [19]. However, this principle requires B/F separation to remove the unbound target from the solution and the addition of a redox probe in the analytical solution. In this study, we focused on the flexibility of the bivalent aptamer structure and employed SWV as the detection method. By application of a redox probe-modified flexible aptamer as the biosensing molecule, we enabled the development of the novel bevacizumab sensor, which does not require a B/F separation, a free redox probe in the analytical solution, or any chemical reaction for detection. This aptamer immobilized electrode showed high sensitivity and selectivity against other human IgG CDRs (Figure 3a,b), and was able to detect the bevacizumab from a 10-fold diluted artificial serum sample containing 53 μM BSA and 8 μM human IgG kappa chain as interferences, and the sensitivity was retained compared with the result in PBS solution. The anti-idiotype aptamer A14#1 has already demonstrated that it was able to bind to bevacizumab in the diluted plasma samples [22,23]. The obstacles to apply the aptamer-based sensor for the detection from blood samples are the presence of a high concentration of protein such as human serum albumin and natural IgGs. The investigation of our sensor in the diluted artificial serum, which contains a high concentration of BSA and human IgG kappa chain, revealed that the sensor can detect the target bevacizumab. Together with the fact that the anti-idiotype aptamer A14#1 keeps the same selectivity and sensitivity in the biological sample, these observations demonstrated that this aptamer-based electrochemical sensor can be applied to the detection from real samples. Therefore, our sensor could be a potential application for POCT-based sensing and, furthermore, the continuous monitoring of bevacizumab.

In this principle, the binding of the target is detected by peak current change depending on the structural change of the aptamer. Although the anti-bevacizumab aptamer A14#1 showed the binding against bevacizumab by both EIS measurement (Figure 1a) and ELONA assay [19], a peak current change was not observed with the addition of the target protein by SWV measurement (Figure S3). We attributed this unexpected observation to the possible structure rigidity of the A14#1 aptamer, which may not provide enough conformation change upon the binding to bevacizumab to change sufficient dynamic movement of the redox probe (PES) access to the electrode. Therefore, we employed the bivalent A14#1 aptamer, which consists of two A14#1 aptamers linked with a 44 mer flexible thymine linker, which is suitable for the distance of the CDRs [19]. The bivalent aptamer was originally constructed to improve its binding affinity without compromising its function [2,19]. However, our SWV results provided evidence for the hypothesis that the unique structure of the bivalent A14#1 aptamer is able to undergo a large conformational change during the binding of two bevacizumab CDR regions. This phenomenon was enough to provide a detectable peak current change in SWV as a result of variable redox probe accessibility on the electrode surface. With the application of PES-bivalent A14#1 to the SWV-based sensor, a clear peak current change, depending on the concentration of the target, was observed.

Therapeutic humanized mAbs are currently the most effective and expected medicines for a wide range of diseases because of their specificity and efficiency. However, there are no POC testing devices which can precisely monitor therapeutic mAb levels, although the therapeutic activity of mAbs is closely related to its concentration in serum. Table 1 summarizes the performance of previously reported bevacizumab detection methods. The most prominent feature of the bevacizumab sensors developed in this study is the time required for detection, which is only 30 min after the addition of the target without any washing steps. Although some sensors previously reported are superior in their sensitivity and dynamic range, they all required several complicated manipulations, such as washing and B/F separation, consequently requiring more time for measurement. Nugue et al. reported that the serum bevacizumab concentration average of 17 patients, which was administrated in the way recommended by Roche-Genentech, had a plateau at 1.43 μM. During this measurement, the lowest value was 0.36 μM and the highest one was 2.76 μM [2]. Our sensor can cover this range using the diluted sample at this time. Therefore, our sensor can be applied to the POC-testing sensor of bevacizumab to meet the requirements of medical devices which are needed to maintain the blood therapeutic mAbs concentrations within a stringent range. Furthermore, since our sensor does not need any large equipment, washing steps, chemical reactions, or free redox probe, it can be applied for continuous monitoring in the future by embedding the patient’s body.

## 4. Materials and Methods

### 4.1. Chemical and Materials

All oligonucleotides were purchased from IDT (Coralville, IA, USA). The sequences of these oligonucleotides are listed in Supplementary Information Table S1.

Sodium chloride, potassium chloride, sodium phosphate dibasic, potassium phosphate monobasic, potassium ferricyanide (III), 6-mercapto-1-hexanol (6-MCH), tris(2-carboxyethyl) phosphine hydrochloride (TCEP), tricine, and sodium acetate were purchased from Sigma-Aldrich Co. LLC (St. Louis, MO, USA). The 2-amino-2-(hydroxymethyl) propane-1,3-diol (Tris) and the human IgG kappa chain was purchased from Fisher Scientific Co. LLC (Pittsburgh, PA, USA). Bevacizumab was purchased from Selleck Chemical LLC (Houston, TX, USA). The amine reactive phenazine ethosulfate (arPES) was kindly donated by Dojindo Molecular Technology, Inc. (Rockville, MD, USA). The sodium hydroxide, sulfuric acid, and absolute ethanol were purchased from VWR (Radnor, PA, USA). The platinum wire was purchased from TANAKA Kikinzoku (Tokyo, Japan). Ag/AgCl (3M NaCl) reference electrodes were purchased from BAS Inc. (Tokyo, Japan). Gold disk electrodes were purchased from CH Instrumental (Austin, TX, USA). The composition of artificial serum is shown in Supplementary Information Table S2. For analysis, 10-fold diluted artificial serum was used. The artificial serum solution was diluted by artificial interstitial fluid (ISF), which is the same composition as an artificial serum but does not contain any protein, but 8 μM of human IgG kappa chain was added as an interference.

### 4.2. Preparation and Operation of Faradaic EIS-Based Bevacizumab Sensor

Bare electrodes were prepared by polishing them with alumina powder, followed by sonication in water and absolute ethanol. The polished electrodes were cleaned electrochemically [29]. Cleaned gold electrodes were dipped into a Tris-HCl buffer (pH 7.4) containing 1 μM TCEP and 0.1 μM aptamer overnight. After this step, electrodes immobilized with aptamer were immersed into a Tris-HCl buffer (pH 7.4) containing 1 mM 6-MCH for 2h. The fabricated electrodes were soaked into phosphate-buffered saline (137 mM NaCl, 2.7 mM KCl, 10 mM Na_2_HPO_4_, 1.8 mM KH_2_PO_4_, pH 7.4) (PBS) solution before measurement.

Faradaic EIS measurements were carried out using 10 mM potassium ferricyanide as a redox probe. All of the electrochemical measurements were performed using the three-electrode system with a platinum counter electrode, Ag/AgCl reference electrode, and a working electrode immobilized with aptamer, and the measurements were carried out using the VSP BioLogic potentiostat (BioLogic, Seyssinet-Pariset, France). The fabricated electrodes were interrogated from 1 Hz to 100 kHz with 10 mV as the amplitude of sinusoidal perturbation and 0.2 V bias potential. To confirm the bevacizumab binding to the immobilized anti-idiotype aptamer A14#1 on the electrode surface, proteins (bevacizumab or human IgG kappa chain) were titrated into a 10 mL reaction chamber. Starting from the lowest concentration, proteins from 1 pM to 10 nM (final concentration) were injected into the reaction chamber. Electrodes were incubated for 30 min with bevacizumab prior to EIS measurement. VSP BioLogic potentiostat calculates impedance and phase angle in real-time from the recorded current measurements, which is output as a Nyquist plot. The resulting data were fit to an equivalent circuit (Randles circuit) using EC-Lab^®^ (BioLogic, Seyssinet-Pariset, France) and analyzed to create calibration curves based on the change in resistance to charge transfer (Rct).

To observe the target-specific signal response, fabricated sensors were incubated with the proteins (bevacizumab, human IgG kappa chain) in 3 mL of PBS solution containing 10 mM potassium ferricyanide. Following the measurement of EIS at 0 M proteins, each concentration of proteins was introduced. Each sample was diluted with PBS solution. The electrodes were incubated for 30 min. This entire process was repeated for each concentration of proteins.

### 4.3. Preparation and Operational Conditions of SWV-Based Bevacizumab Sensor Using PES-Modified Aptamer

The amine-modified aptamer (100 µM) was mixed with arPES in a 400 mM tricine buffer (pH 8.0) and incubated at 25 °C overnight. After incubation, PES-modified aptamer was purified from the mixture by ethanol precipitation [30]. The cleaned gold electrodes, prepared as described above, were dipped into a Tris-HCl buffer (pH 7.4) containing 1 μM TCEP and a 0.1 μM PES-modified aptamer overnight. After this step, electrodes immobilized with an arPES-modified aptamer were immersed into a Tris-HCl buffer (pH 7.4) containing 1 mM 6-MCH for 2 h. The fabricated electrodes were soaked in a PBS solution before measurement.

Following the measurement of SWV at 0M, 1–100 nM of bevacizumab was titrated into electrochemical analysis cells. Electrodes were incubated for 30 min before measuring the SWV. The fabricated sensors were interrogated from −0.1 to −0.4 V (vs. Ag/AgCl) with 25 mV amplitude, 4 mV steps, and 5 to 250 Hz frequency range. The obtained voltammograms were smoothed by Savitsky-Golay filters.

## 5. Conclusions

In this study, by focusing on the anti-idiotype aptamer as the biosensing molecule for therapeutic mAb, bevacizumab, an electrochemical aptamer-based sensor was developed. Our biosensor, which can be integrated into a small handheld electrochemical device, can monitor the concentration of bevacizumab within 30 min by employing an anti-idiotype bivalent A14# aptamer following the SWV principle. The SWV-based bevacizumab sensor with redox probe-modified bivalent A14#1 achieved the detection of bevacizumab from 1–100 nM, and the LOD was 9.1 nM in PBS solution. These sensors effectively distinguished the target IgG, bevacizumab, from another natural human IgG. The feasibility of monitoring in a biologically relevant sample matrix was also demonstrated through the detection of bevacizumab in a 10-fold diluted artificial serum solution, where we achieved a LOD of 10.1 nM, covering the physiologically relevant concentration range (1–100 nM). This study demonstrated the development of electrochemical sensors for a therapeutic monoclonal antibody that can perform easily and rapidly, and we hope that these insights will contribute to the development of a continuous monitoring system for therapeutic mAbs in order to improve patient outcomes.

## Figures and Tables

**Table 1:** Comparison of previously reported sensing principles for bevacizumab and this work.

Measurement Principle	Probe	Time	Range	LOD	Ref
enzyme-linked immunosorbent assay (ELISA)	VEGF	6 h	34–500 nM	0.22 nM	[6]
Immunoassay with a suspension array	VEGF	2 h	34–500 nM	-	[2]
HT-RPLC with antibody	Antibody	>2 h	6.7–133 nM	0.46 nM	[26]
Proximity extension assay	Antibody		1–1000 pM	1 pM (LLOQ)	[27]
Enzyme-linked aptamer assay (ELAA)	Aptamer	>4 h	340 pM–34 nM	14 pM	[22]
HT-RPLC with aptamer	Aptamer	>2 h	6.7–340 nM	1 nM	[23]
Liquid chromatography-based multiple reaction monitoring MS	-	>6 h		12 nM	[28]
Electrochemical detection with free redox probe	Aptamer	>160 min	31-500 pM	0.1 nM	[19]
Voltammetry	Aptamer	30 min	1–100 nM 1–100 nM	9.1 nM (PBS) 10.1 nM (artificial-serum)	This study

## Data Availability

Any data or material that support the findings of this study can be made available by the corresponding authors upon request.

## Supplementary Materials

The following supporting information can be downloaded at: https://www.mdpi.com/article/10.3390/ijms24065277/s1.
