# Supplementary material for: Development of an Anti-Idiotype Aptamer-Based Electrochemical Sensor for a Humanized Therapeutic Antibody Monitoring
Source: Int J Mol Sci. 2023 Mar 9;24(6):5277. doi: 10.3390/ijms24065277 (PMC10048959; doi:10.3390/ijms24065277)
Supplement: Supplementary file 1 [file ijms-24-05277-s001.zip › ijms-2242364-supplementary.pdf]

Supporting Information

Development of electrochemical sensors for a humanized antibody, bevacizumab, employing anti-idiotype aptamers

Madoka Nagata[1, 2], Jinhee Lee[1], Taro Saito[2], Kazunori Ikebukuro[2], and Koji Sode[1]*

Table S1 DNA Sequences against bevacizumab.

Name	Sequence (5' > 3')	Length
A14#1	GCGGTTGGTGGTAGTTACGTTCGC	24
BivA14#1	GCGGTTGGTGGTAGTTACGTTCGCTTTTTTTTTTTTTTTTTTTTT<br>TTTTTTTTTTTTTTTTTTTTTTTGCGGTTGGTGGTAGTTACGTTC<br>G	92

Table S2 Composition of artificial serum solution.

Artificial serum composition	pH 7.44
NaCl	123 mM
KCl	3.5 mM
$CaCl_2$	2.5 mM
$MgCl_2$	0.7 mM
NaH_2PO_4	1.5 mM
HEPES	10 mM
Bovine serum albumin	533 μM

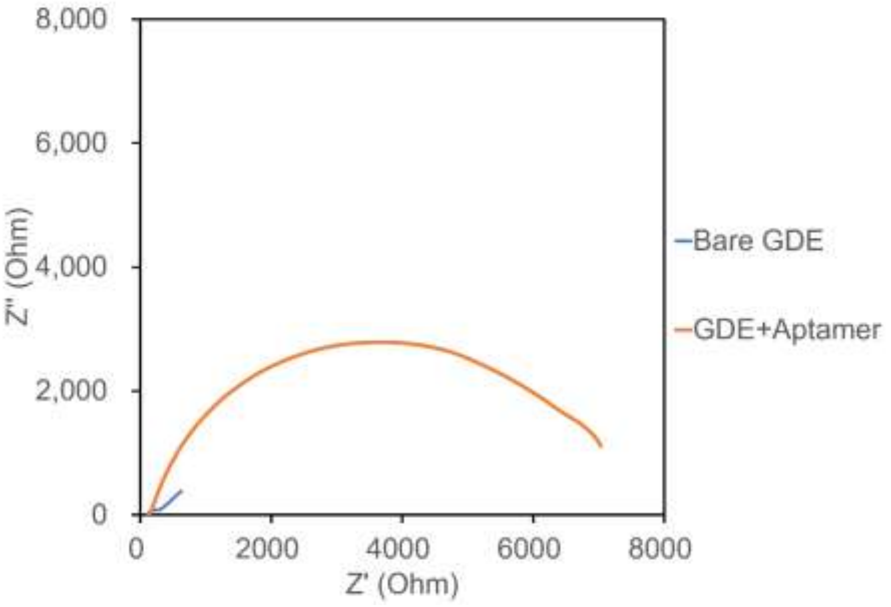

Figure S1 The Nyquist plots before and after the modification of aptamer

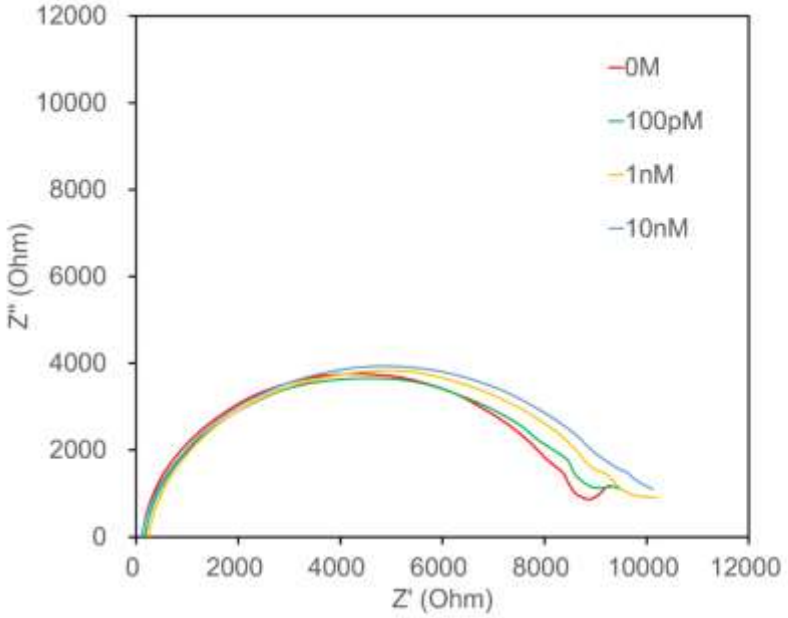

Figure S2 EIS monitoring of bevacizumab using anti-bevacizumab aptamer A14#1 immobilized electrode with the addition of human IgG kappa chain.

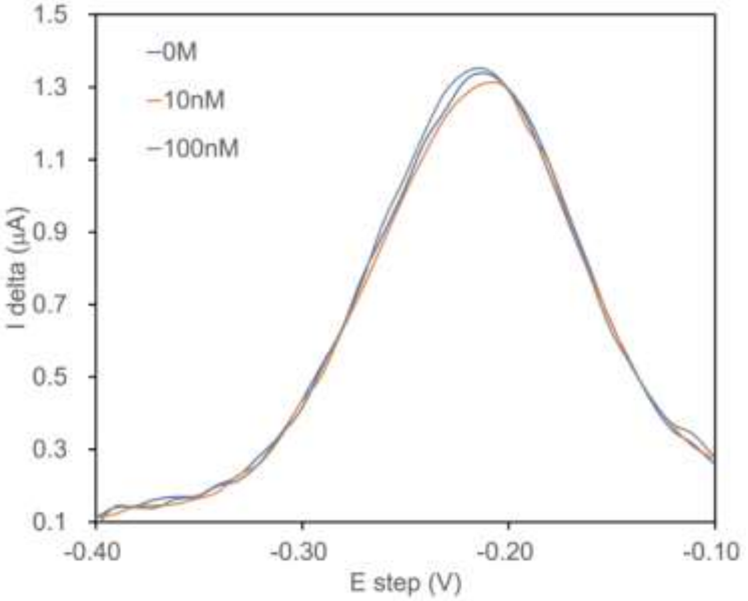

Figure S3 Square-wave voltammogram of PES-monovalent A14#1 aptamer immobilized sensor at 100Hz in PBS solution with the addition of several concentrations of bevacizumab. SWV scans were conducted as below conditions: -0.4 to -0.1 V, 25 mV amplitude.

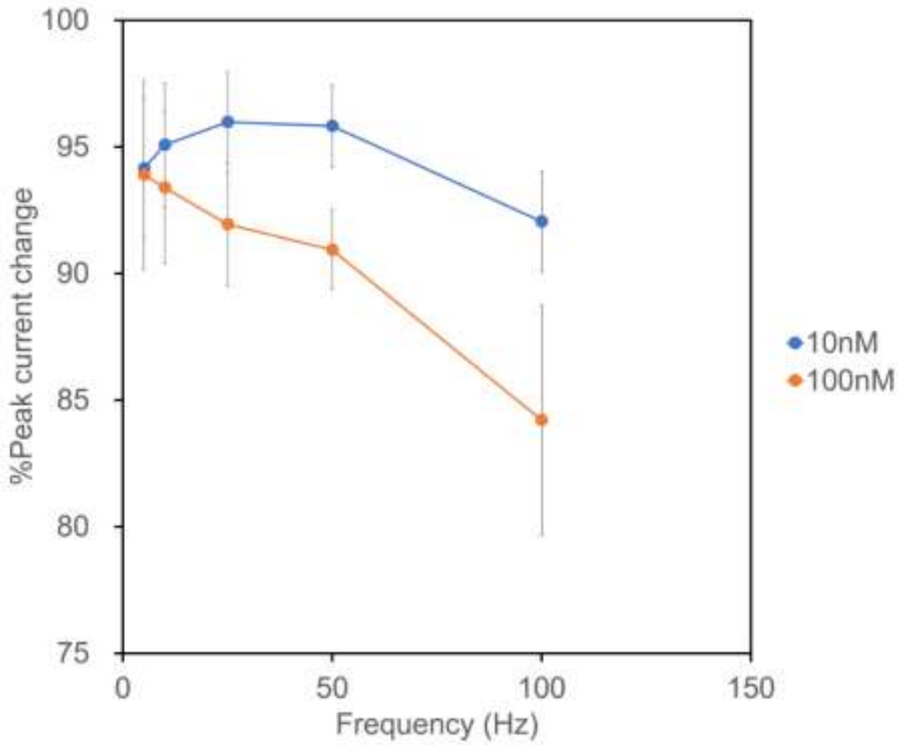

Figure S4 Peak current change dependencies on the frequency at each bevacizumab concentration. The error bars indicate the standard deviations (N=3).
